# Management Based on Pretreatment PSMA PET of Patients with Localized High-Risk Prostate Cancer Part 2: Prediction of Recurrence—A Systematic Review and Meta-Analysis

**DOI:** 10.3390/cancers17050841

**Published:** 2025-02-28

**Authors:** Manuela Andrea Hoffmann, Cigdem Soydal, Irene Virgolini, Murat Tuncel, Kalevi Kairemo, Daniel S. Kapp, Finn Edler von Eyben

**Affiliations:** 1 Department of Nuclear Medicine, University Medical Center of the Johannes Gutenberg University, 55131 Mainz, Germany; manhoffm@uni-mainz.de; 2 Institute for Preventive Medicine of the German Armed Forces, 56626 Andernach, Germany; 3 Department of Nuclear Medicine, University of Ankara, Ankara 06050, Turkey; csoydal@yahoo.com; 4 Department of Nuclear Medicine, University Hospital Innsbruck, 6020 Innsbruck, Austria; irene.virgolini@tirol-kliniken.at; 5 Department of Nuclear Medicine, Hacettepe University, Ankara 06800, Turkey; murat.tuncel@hacettepe.edu.tr; 6 Docrates Cancer Center, 00180 Helsinki, Finland; kalevi.kairemo@gmail.com; 7 Department of Radiation Oncology, Stanford University, Stanford, CA 94305, USA; dskapp@stanford.edu; 8 Center of Tobacco Control Research, 5230 Odense, Denmark

**Keywords:** prostate neoplasms, prostate specific membrane antigen PET, recurrence, survival

## Abstract

Background and objectives: For patients with prostate cancer (PCa), PSMA PET better diagnose metastases than conventional imaging. In a systematic review and meta-analysis (INPLASY register, 2024311004), we aimed to summarize findings with pretreatment PSMA PET in patients with PCa that was localized according to conventional imaging and summarize how pretreatment PSMA PET had influence on biochemical recurrence (BCR)-free survival and overall survival (OS). Methods: We searched for publications in Pubmed, Google Scholar, ClinicalTrials.gov, and reference lists between 2016 and February 2025. We summarized biochemical recurrence-free survival in Forest plots. Results: Nine publications reported 1908 patients and showed that pretreatment PSMA PET was associated with survival. Three publications reported that pretreatment PSMA PET gave better 3–5-year BCR-free survival than conventional imaging (74% versus 57%). Two publications reported PSMA PET-risk for 389 patients. Those with PSMA PET-low-risk lived 5 years longer often than those with PSMA PET high-risk (84% versus 20%). Conclusions: Pretreatment PSMA PET is widely used in the real world. Pretreatment PSMA PET supports personalized treatment and may explain why pretreatment PSMA PET improved BCR-free survival and OS. It is believed that pretreatment PSMA PET may facilitate future progress in care of patients with high-risk PCa.

## Simple Summary

Pretreatment PSMA PET at diagnosis is widely used and treatment changes based on its findings gave better biochemical recurrent (BCR)-free survival than conventional imaging. The findings is a strong argument in favor of using PSMA PET not only for patients with biochemical recurrence but also for patients undergoing primary staging. The use of pretreatment PSMA PET is wide-spread but the clinical benefit indicated in the systematic review has not been confirmed in a prospective randomized trial.

## 1. Introduction

Identification of patients for evaluation of prostate cancer (PCa) starts with men having symptoms or a raised prostate-specific antigen (PSA). After referral to a prostate cancer clinic or other health care providers, the evaluation includes an ultrasound, an MRI, and a diagnostic biopsy. Patients with localized PCa by conventional staging are followed with surveillance or undergo focal treatment, radical prostatectomy (RP), or external beam radiotherapy (EBRT) or brachytherapy. After primary treatment with a curative intention, a quarter of the patients have biochemical recurrence (BCR) [1]. The risk of recurrence is increased for PCa patients who express prostate-specific membrane antigen (PSMA). Patients with aggressive PCa have an especially high uptake of PSMA PET in PCa lesions [2].

[^68^Ga]Ga-PSMA-PET/CT diagnoses metastases better than conventional imaging [3]. The Federal Drug Administration (FDA) approved three PSMA imaging agents [3,4,5]: [^68^Ga]Ga-PSMA(gozetotide, Illuccix^®^, and Locametz^®^), [^18^F]F-PSMA DCF Pyl (Piflufolast), and [^18^F]F-rh-PSMA-7 PET. Also, [^18^F]F-PSMA-1007 is used increasingly [6,7]. Today, many high-risk (HRPC) patients are staged with pretreatment PSMA PET [8]. But oncologists disagree on treatment of metastases diagnosed only on pretreatment PSMA PET. In 2024, the European Association of Urology (EAU) postponed recommending pretreatment PSMA PET, pending studies showing a survival benefit.

To elucidate the issue, we undertook a systematic review and meta-analysis (SR and MA, INPLASY register number 202410061). The aim was to summarize the pretreatment impact on BCR-free survival and overall survival (OS). Complementarily, we published a SR on the impact pretreatment PSMA PET had on the primary staging and treatment.

## 2. Methods

### 2.1. Materials and Methods

During the period August to February 2025, we undertook a SR according to the guidelines of the Preferred Reporting Items for Systematic Analysis (PRISMA) [9]. In the previous SR, Hoffman et al. [10] described the search terms for our search of literature. This current SR included original research publications published in English from 2016 to February 2025 on patients with localized PCa by conventional imaging who had undergone PSMA PET where the publications reported BCR-free survival and OS. The target population was patients with intermediate risk (IRPC) and HRPC who had localized PCa by conventional imaging, had undergone pretreatment PSMA PET, and had been followed for BCR and OS. The intervention test was pretreatment PSMA PET, and the control test was conventional imaging. The outcomes were BCR-free survival and OS.

### 2.2. Definitions

PCa pathology was redefined by the International Society of Urologic Pathology (ISUP) 2019 [11]. Intraprostatic lesions with ISUP 1 were defined as insignificant whereas lesions with ISUP ≥ 2 were defined as significant. Micrometastases and PSA were defined as previously reported [10]. Risk was defined by the classification of D’Amico et al. [12].

Performance and reporting of PSMA PET employed the Prostate Cancer Molecular Imaging Standardized Evaluation Framework Including Response Evaluation version 2 (PROMISE V2) [13]. Conventional staging includes digital rectal exploration (DRE), ultrasound, CT, and bone scans. Based on conventional staging, lesions in the prostate, lymph nodes, and bones were defined as cT1, cN1, and cM1, respectively. Similarly, based on pretreatment PSMA PET, positive sites in the three regions were defined as miT1, miN1, and miM1 [13], while findings based on pathology were defined as pT1, pN1, and pM1. BCR after radiation therapy was defined by the Phoenix criteria [14].

Whole-body PSMA PET was defined as a scan from the skull base to the mid-thigh. Uptake time for the PET tracer, standard uptake value (SUV), and positive findings were defined as previously published [10].

Since extrapelvic lymph node metastases (LNM) behaved clinically more like pelvic LNM than like metastases in other distant organs, we included extrapelvic LNM as LNM. Patients with metastases in both lymph nodes and bones were included as bone metastases (BMs), as were patients with metastases both in bones and viscera. At diagnosis, very few patients have visceral metastases.

Downstage by PSMA PET was defined as a lower stage on PSMA PET than on conventional imaging. Upstage by PSMA PET was defined as a higher stage on PSMA PET than on conventional imaging. Extended pelvic lymph node dissection (ePLND) was defined as dissection of lymph nodes in the regions of the internal and external iliac vessels up to the bifurcation of the common iliac artery and the obturator fossa.

Biochemical persistence was defined as PSA remaining > 100 ng/L 4–8 weeks after RP. Oligometastatic PCa was defined as PSMA PET diagnosing up to five positive sites. For patients treated with RP, BCR was defined as a reduction in PSA to unmeasurable values, followed by a rise in PSA in three measurements taken with at least one week interval.

### 2.3. Statistical Analysis

We summarized both median and mean values as median values. Publications of BCR-free survival and OS after pretreatment PSMA-PET were summarized in Forest plots by the methods of Nyaga et al. [15]. A *p* value < 0.05 was defined as statistically significant. All statistical analyses were carried out using STATA version 14 with updates (STATA Corp., College Station, TX, USA).

## 3. Results

### 3.1. Selection of Publications

**Figure 1:**
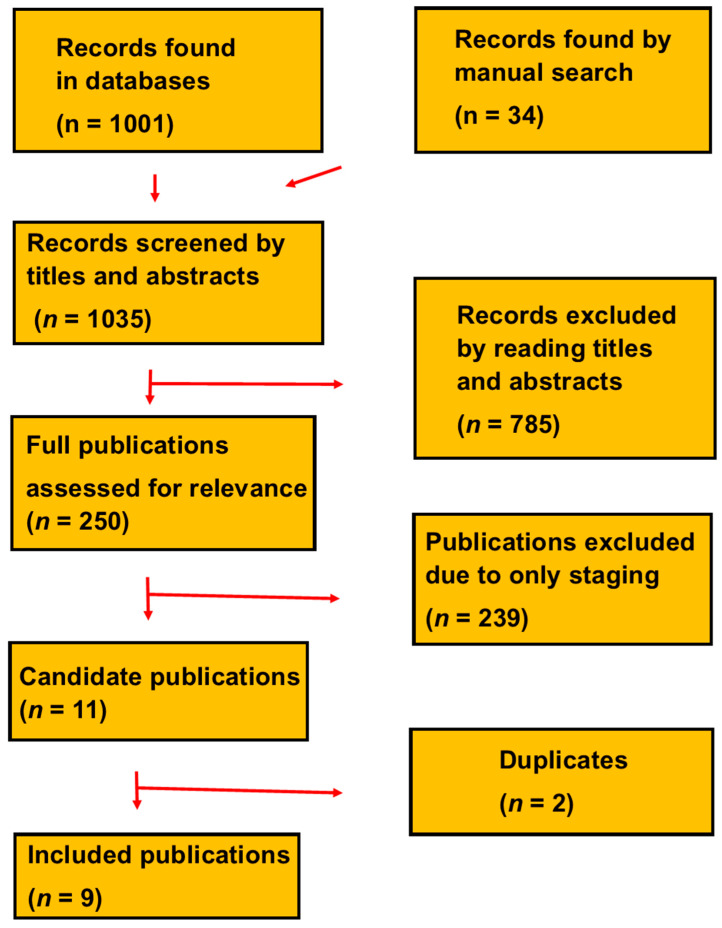
PRISMA flow chart of how we selected the nine publications.

We identified nine original research publications that reported survival, as shown in Figure 1 [16,17,18,19,20,21,22,23,24]. The publications included 1908 patients. Table 1 shows clinical characteristics. The patients had been examined at radiologic centers between 2007 and December 2021.

### 3.2. PSMA PET Methods

PSMA methods for the publications are shown in Table 2. Nuclear medicine physicians who evaluated the pretreatment PSMA PET and multidisciplinary teams for the management of the patients had access to all clinical information and the results of the conventional imaging.

### 3.3. Diagnostic Performance of Pretreatment PSMA PET

Hoffmann et al. [10] found that pretreatment PSMA diagnosed LNMs for 22% of the patients and bone metastases for 16% of them. Isolated increased activity in scapula, positive findings in abdominal ganglions, and other benign and malignant disorders are likely to be false-positive [25]. In contrast, a SUV_max_ > 10 is most often true positive [26]. PSMA PET does not detect most small cell and neuroendocrine PCa [27,28]. Hoffmann et al. [10] reported the rates of false-positive and false-negative pretreatment PSMA PET estimated by analyses of LNM after ePLND. Rate of false-negative findings were nearly as frequent as true positive findings (13% versus 14%) because PSMA PET rarely detect LNM with a diameter < 4 mm.

### 3.4. Change in the Primary Stage and Treatment

Pretreatment PSMA PET can change the stage defined by conventional imaging. The SR by Hoffmann et al. [10] found that pretreatment PSMA downstaged 19% and upstaged 22%. The change in stage by pretreatment PSMA PET motivated a change in the treatment that was planned based on conventional imaging.

Shakespeare et al. [29] found that patients with LNM given PSMA PET-guided volumetric radiation therapy (VMAT) had an excellent 2-year survival free of recurrence. Patients who had oligometastatic PCa diagnosed by pretreatment PSMA PET can be treated with metastasis-directed therapy (MDT) with or without systemic combination therapy [30]. Patients with polymetastatic PCa diagnosed by pretreatment PSMA PET should be treated with systemic combination therapy [31]. However, many centers initially treat patients with metastatic hormone-sensitive PCa with ADT monotherapy, despite a trend for doublet and triplet treatment.

### 3.5. PSMA PET and the Initial Treatments

Some centers use per-operative PSMA PET to localize and dissect PSMA-positive lymph nodes [32]. Regarding radiation therapy, patients had better tumor control and developed less local recurrence if a focal radiation boost targeting DIL was added to the EBRT of the whole prostate than patients treated with only the EBRT [33]. Most radiation oncologists used mpMRI to delineate DIL, but it has also been delineated with PSMA PET [34]. The FLAME trial [35] showed that it was important that the EBRT for the prostate included a boost to the DIL. Half of German radiation oncologists used pretreatment PSMA PET for the radiation therapy [36].

Van Leeuwen et al. [37] found that LNM diagnosed with pretreatment PSMA PET highly predicted PSA persistence, whereas it was not predicted by stage and PCa present at the surgical margins of RP.

### 3.6. PSMA PET After the Initial Treatment

After the primary treatments, repeat PSA measurements monitored the patients, but restaging PSMA PET was also used [38]. Kleiburg et al. [39] found that the PSMA PET response predicted OS better than the PSA response.

### 3.7. Radioligand Therapy

PSMA PET identifies patients who might benefit from a theranostic approach. PSMA PET-positive patients may gain from PSMA-based radioligand therapy (PRLT). Two RCTs (TheraP and VISION) studied third-line treatment of patients with metastatic castration-resistant PCa (mCRPC) [40,41]. PRLT increased the PSA response relative to the comparator treatment. The Enza-P RCT [42] showed that PRLT also was effective as first-line treatment. Furthermore, the LUTectomy study [43] found that neoadjuvant PRLT monotherapy had positive effects. Also, Golan et al. [44] used PRLT as neoadjuvant monotherapy.

### 3.8. Survival

**Figure 2:**
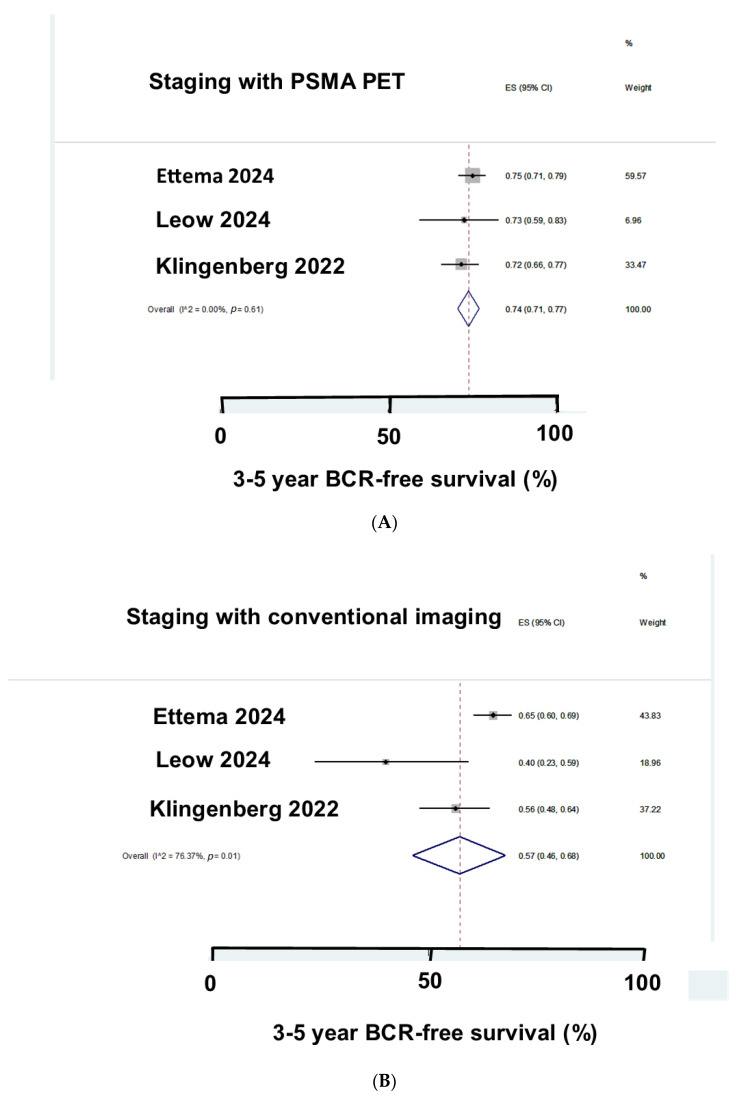
BCR-free survival in three studies [16,18,21]. Staging with PSMA PET (**A**) gave a better BCR-free survival than staging with conventional imaging (**B**).

Ettema et al. [17] reported the 3-year BCR-free survival after staging with PSMA PET or conventional imaging. Patients staged with PSMA PET had a 75% 3 yr BCR-free survival, whereas it was 65% for the patients staged with conventional imaging. Leow et al. [19] studied clinically node positive patients staged with pretreatment PSMA PET or conventional imaging. Patients staged with pretreatment PSMA PET had a 72% 4-year BCR-free survival, whereas it was 38% for the patients staged with conventional imaging. Klingenberg et al. [22] reported HRPC patients. In total, 247 patients had been staged with PSMA PET and 137 had been staged with conventional imaging. Treatment based on PSMA-PET markedly increased the 5-year BCR-free survival compared with treatment based on conventional imaging (71.2% versus 56.4%, HR = 0.58, *p* = 0.004). Figure 2 shows Forest plots of the three studies. Pretreatment PSMA PET gave a better 3–5-year BCR-free survival than conventional imaging (74% versus 57%). Pretreatment PSMA PET markedly reduced the risk of BCR (HR = 0.60).

Bauckneht et al. [16] studied the impact of changes in staging with pretreatment PSMA PET. After 1 year, downstaged patients and patients with unchanged stage had a higher BCR-free survival than the upstaged patients (80% versus 52%). Roberts et al. [23] found that a high SUV_max_ in the primary tumor by PSMA PET predicted subsequent progression-free survival. Patients with ISUP ≤ 3 had a better 3-year progression-free survival than patients with ISUP 4–5 (92% versus 65%). Meijer et al. [20] studied 493 patients with IRPC or HRPC. In total, 70% of the patients with lymph nodes negative for metastases on pretreatment PSMA PET (miN0) lived for 2 years without biochemical progression, in contrast to only 20% of the patients with lymph nodes that were positive for metastases on the pretreatment PSMA PET (miN1). In multivariate logistic regression analysis, Gleason score, extracapsular extension of PCa on mpMRI. and LNM on PSMA PET (miN1) predicted biochemical progression. Marra et al. [20] studied patients with LNM and found that the PSMA PET-positive patients progressed more often than the PSMA PET-negative patients (53% versus 14%).

In the ORIOLE trial of patients with BCR [45], 95% of the patients who were treated for all positive lesions on PSMA PET lived 6 for months without progression, in contrast to only 62% of the patients who had at least one untreated lesion (HR = 0.25, *p* = 0.006).

**Figure 3:**
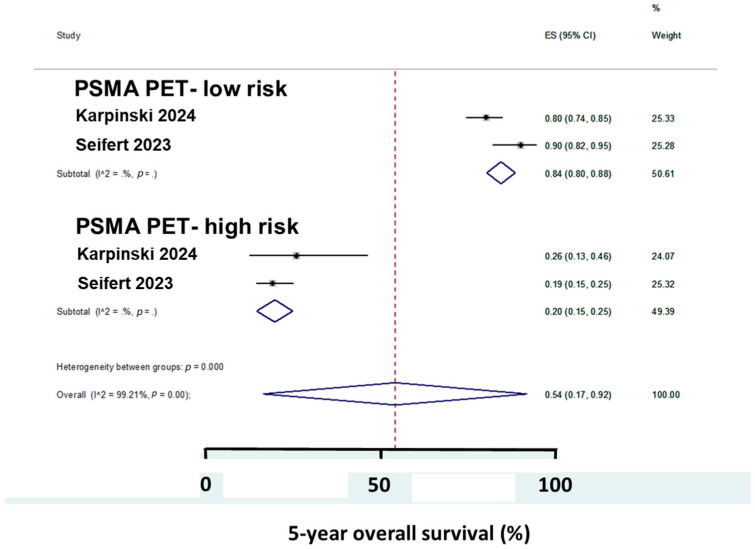
The 5-year overall survival in two studies with 389 patients according to PSMA PET-risk groups [17,20].

Two studies [18,21] showed that risk groups classified by PSMA-PET had different 5-year OS (84% vs. 20%), as shown in Figure 3.

## 4. Discussion

Pretreatment PSMA PET enabled a better personalized treatment because pretreatment PSMA PET for patients with HRPC showed that a third of the patients initially had metastatic PCa, whereas conventional imaging only found localized cancer [10]. The more sensitive pretreatment PSMA PET improved BCR-free survival and OS. The PSMA PET-low-risk group of patients had an 84% 5-year OS, whereas it was markedly lower for the PSMA PET-high risk group.

Oncologists may prefer to treat metastases diagnosed only on pretreatment PSMA PET than to treat the metastases later when they are diagnosed with conventional imaging. In the real world, physicians have implemented pretreatment PSMA PET more often than several international guidelines recommended. But long-term follow-up is needed to fully document the survival advantages of treatment based on pretreatment PSMA PET. Pretreatment PSMA PET for HRPC patients (which is not generally recommended) seems to predict OS better than ePLND (which is recommended by EAU).

The literature on survival after pretreatment PSMA PET was more extensive than previous guidelines and reviews had summarized. But, at present, the literature on OS after pretreatment PSMA PET is limited. Early treatment may treat patients in a clinical phase before the cancer has developed secondary mutations and progressive heterogeneity. A combination of pretreatment fluorodeoxyglucose (FDG) and PSMA PET can help nuclear medicine physicians to point out if PCa lesions have heterogeneous expression of PSMA [38].

Pretreatment PSMA PET is the best imaging modality to diagnose oligometastatic PCa among patients with metastatic PCa. For those with oligometastatic PCa, MDT should target all metastases.

Pretreatment PSMA PET leads to better BCR-free survival and OS than conventional imaging because downstaging with pretreatment PSMA PET scans motivates targeted curative loco-regional treatment as an alternative to palliative systemic treatment. Upstaging with pretreatment, PSMA PET motivates systemic and metastasis-directed treatment as an alternative to standard loco-regional treatment. It remains to elucidate how often the personalized approaches are adopted.

Pretreatment PSMA PET uses well-documented and approved methods. The attractive perspectives reported with the PET scans can be tested. PCa has a long tradition for investigating treatments that were effective in advanced disease, early in the clinical course, as recommended by the Prostate Cancer Clinical Trialists Working Group 3 (PCWG3) [46].

There is a tumor biologic chain from aggressive, partly de-differentiated PCa over high expression of PSMA, to metastatic spread and reduced survival. Staging with only conventional imaging prohibits decisions regarding PRLT, in contrast to a positive pretreatment PSMA PET. Positivity is the key criterion for using PRLT.

Early use of PRLT and the combination of ARPI and PRLT are more effective than the PRLT monotherapy for patients with mCRPC. However, the literature on pretreatment PSMA PET has ignored this approach.

Small studies have shown promising results with neoadjuvant PRLT and ARPI as monotherapies [41,42]. But, so far, the literature on neoadjuvant treatment of PCa is limited. Many centers still treat patients with metastatic hormone-sensitive PCa with ADT monotherapy, despite evidence from ARASENSE and ARANOTE trials that doublet or triplet therapy yields better responses [47,48]. It remains to be seen if early treatment of metastases diagnosed with pretreatment PSMA PET may improve PCa-specific survival.

It should be noted that the benefits of pretreatment PSMA PET compared with conventional imaging are also present with choline PET. However, PSMA PET is more sensitive for PCa patients with low PSA than choline PET [49]. So, most centers prefer to stage the patients with PSMA PET rather than choline PET.

For breast carcinoma with regional LNMs, adjuvant systemic therapy following primary surgery of the breast using effective treatment for advanced breast cancer provides a survival benefit [50]. It remains to be shown whether patients with HRPC obtain a similar survival benefit from adjuvant treatment.

As a strength, our SR and MA selected publications published after 2015 to reduce treatment bias. Judged by the Quality Assessment of Diagnostic Accuracy in Studies version 2 (QUADAS2) [51], the quality of the publications was high. For pretreatment PSMA PET, the literature documented a better effect regarding BCR-free survival compared to OS. A limitation is that most publications were retrospective. A second limitation is that our review did not address restaging PSMA PET of patients with BCR and metastatic PCa. However, metastatic findings of pretreatment PSMA PET closely correspond with those of restaging PSMA PET in cases of progressing PCa. A third limitation is that progress in other aspects PCa management may also improve patient survival.

## 5. Conclusions

Pretreatment PSMA PET supports personalized treatment of patients with HRPC and explains why changes in treatment based on the scans may improve BCR-free survival and OS. However, RCTs are needed to evaluate whether these encouraging results can be reproduced in controlled settings. It is believed that PSMA PET may facilitate future progress for patients with HRPC.

## Figures and Tables

**Table 1:** Characteristics in publications of pretreatment PSMA PET followed for recurrence and survival.

Author, Year	Type of Study	Study		No Pts	Median Age (Years)	Rate with ISUP 4/5	Median PSA (ng/mL)
		Start	End				
Roberts et al., 2021 [24]	R	2014	2017	71	64	32	7.4
Klingenberg et al., 2022 [22]	R	2016	2019	247	NR	32	NR
Meijer et al., 2022 [23]	R	NR	NR	409	68	33	93
Seifert et al., 2023 [21]	R	2014	2018	164	70	24	182
Bauckneht et al. [16]	R	2014	2021	97	67	65	16.8
Ettema et al., 2024 [17]	R	2016	2021	440	67	43	9.8
Karpinski et al., 2024 [18]	R	2014	2021	225	69	NR	9.1
Leow et al., 2024 [19]	P	2007	NR	76	74	64	14
Marra et al., 2024 [20]	R	2016	2021	95	66	NR	11.1

Abbreviations: NR not reported, P prospective study, R retrospective study.

**Table 2:** PSMA PET characteristics.

Author, Year	Tracer			CT/MRI	Median Activity (MBq)	Median Uptake Time (min)		
	68Ga	18F DCF	18F 1007			68Ga	18F DCF	18F 1007
Roberts et al., 2021 [24]	X	-	-	CT	NR	45	-	-
Klingenberg et al., 2022 [22]	X	-	-	CT	214	60	-	-
Meijer et al., 2022 [23]	X	X	X	CT	103	45	117	90
Seifert et al., 2023 [21]	X	-	-	CT	NR	60	-	-
Bauckneht et al., 2024 [16]	X	-	X	CT	NR	NR	-	NR
Ettema et al., 2024 [17]	X	X	X	CT	NR	50	119	90
Karpinski et al., 2024 [18]	NR	NR	NR	CT/MRI	NR	NR	NR	NR
Leow et al., 2024 [19]	NR	NR	NR	NR	NR	NR	NR	NR
Marra et al., 2024 [20]	NR	NR	NR	NR	NR	NR	NR	NR

Abbreviations: NR: not reported, X: used, -: not used.

## Abbreviations

ADT	Androgen deprivation therapy
ARPI	Androgen receptor pathway inhibitor
BCR	Biochemical recurrence
BM	Bone metastases
CT	Computed tomography
DRE	Digital rectal exploration
DIL	Dominant intraprostatic lesion
EBRT	External beam radiation therapy
ePLND	Extensive pelvic lymph node dissection
F	Fluoride
FDA	Federal Drug Administration
Ga	Gallium
HRPC	High-risk prostate cancer
IRPC	intermediate risk prostate cancer
ISUP	International Society of Pathologists
LNM	Lymph node metastases
LRPC	Low-risk prostate cancer
M	Metastasis (distant)
MA	Meta-analysis
mCRPC	Metastatic castration-resistant prostate cancer
MDP	Methylene diphosphonates
mpMRI	Multiparametric magnetic resonance imaging
N	Lymph nodes
OS	Overall survival
PCa	Prostate cancer
PCWG3	(4) Prostate cancer trial working group 3 (4)
PET	Positron emission tomography
PRISMA	Preferred reporting Items for Systematic Reviews
PRLT	PSMA based radioligand therapy
PROMISE	v2 Prostate Cancer Molecular Imaging Standardized Evaluation Framework Including Response Evaluation version 2
PSA	Prostate specific antigen
PSMA	Prostate specific membrane antigen
QUADAS	2 Quality Assessment of Diagnostic Accuracy in Studies version 2
RCT	Randomized controlled trial
RP	Radical prostatectomy
SR	Systematic review
SUV	Standard uptake value
T	Primary tumor
Tc	Technetium
